# Whole‐Food Protein Sources Differentially Modulate Postprandial Plasma Dileucine Profiles in the Post‐Exercise State

**DOI:** 10.1002/mnfr.70628

**Published:** 2026-10-19

**Authors:** Žan Zupančič, Geoffrey M. Provenzano, Calvin Chen, Gena L. Irwin, Alexander V. Ulanov, Nicholas A. Burd

**Affiliations:** ^1^ Department of Health and Kinesiology University of Illinois Urbana‐Champaign Urbana Illinois USA; ^2^ Institute of Neuroscience Université Catholique De Louvain Louvain‐La‐Neuve Belgium; ^3^ Roy J. Carver Biotechnology Center University of Illinois Urbana‐Champaign Urbana Illinois USA; ^4^ Division of Nutritional Sciences University of Illinois Urbana‐Champaign Urbana Illinois USA

**Keywords:** animal protein, dileucine, leucine, plant protein, skeletal muscle, whole foods

## Abstract

Dileucine, a leucine‐containing dipeptide, may enhance anabolic signaling more than leucine alone. This study examined postprandial plasma dileucine and leucine concentrations after ingesting animal‐ and plant‐ whole foods and their association with muscle protein synthesis rates (MPS). Secondary analysis was performed on plasma samples collected from two randomized controlled trials totaling 26 healthy adults (25 ± 4.3 y). Participants consumed low‐fat pork (LFP), high‐fat ground pork (HFP), complementary rice and beans (COMP), an iso‐nitrogenous mixed nutrient beverage (ISO), or a carbohydrate control (CHO). Plasma concentrations and MPS was measured. Plasma dileucine increased significantly only after LFP ingestion (*p* < 0.05), with a greater 300‐min iAUC compared to CHO (*p* = 0.038). Plasma leucine increased in all conditions except COMP and CHO with LFP demonstrating the highest concentrations (*p* < 0.05). A modest correlation was observed between dileucine C*max* and the change in MPS (ΔMPS; r = 0.436, *p* = 0.037), whereas leucine C*max* showed no significant association (r = 0.265, *p* = 0.102). Whole‐food protein sources generally do not elevate plasma dileucine, except for LFP. While dileucine C_max_ correlated with ΔMPS, the strength of this relationship suggests dileucine is likely one of several postprandial factors influencing MPS beyond free leucine.

## Graphical abstract

This study examined how consuming various foods during recovery from resistance exercise affects plasma dileucine concentrations, a dipeptide potentially involved in stimulating muscle protein synthesis rates (MPS). Among the foods tested, only ground low‐fat pork (LFP) ingested immediately after resistance exercise induced a significant increase in circulating dileucine. Furthermore, higher peak concentrations of plasma dileucine, but not leucine, were directly associated with increased MPS. These findings demonstrate that a food's structure and digestion rate are regulating variables for the postprandial release of muscle‐building nutrients into circulation in healthy young adults.

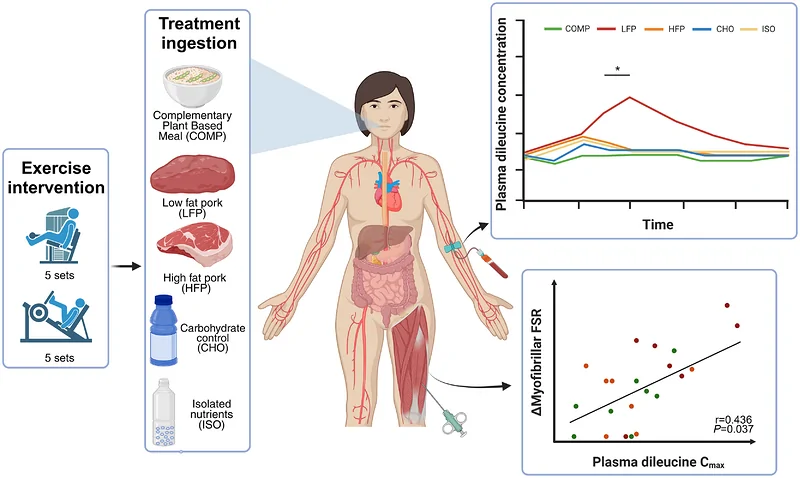

**Abbreviations**
CHO: carbohydrate control drink
COMP: complementary pairing of rice and black beans
Cmax: peak plasma amino acid concentration
DILEU: dileucine
EAA: plasma essential amino acids
FSR: fractional synthesis rate
GSLTPAQ: Godin‐Shephard Leisure‐Time Physical Activity Questionnaire
HFP: high fat pork
iAUC: incremental area under the curve
IPAQ: International Physical Activity Questionnaires
ISO: isolated mixture of crystallized amino acids, maltodextrin, cellulose, pectin, and soy oil.
LFP: low fat pork
MPS: muscle protein synthesis
Tmax: time of peak plasma amino acid concentration
ΔMPS: change in myofibrillar protein synthesis rates from baseline

## 1 Introduction

Dietary protein ingestion stimulates postprandial increases in circulating amino acids, which serve as both substrates and signaling molecules for the regulation of skeletal muscle protein synthesis (MPS) [1, 2, 3, 4]. Among essential amino acids, leucine has been identified as a key regulator of anabolic signaling through activation of the mechanistic target of rapamycin complex 1 (mTORC1), contributing to the stimulation of MPS in both resting [5] and post‐exercise conditions [6, 7]. Beyond amino acids, dietary proteins also contain small peptides generated during digestion, including dipeptides and tripeptides, which may enter the circulation intact. Specifically, di‐ and tripeptides are transported across the intestinal epithelium via PepT1 H^+^, a proton‐coupled peptide transporter located on the apical membrane of enterocytes [8]. Importantly, Pept1 functions as a high‐capacity and low‐affinity transporter that allows for the rapid clearance of large concentrations of intact‐short‐chain peptides into the enterocyte [8].

Among food‐derived peptides, the leucine‐containing dipeptide dileucine (Leu–Leu) has received increasing attention due to the established anabolic role of leucine. Recent work using a crossover design demonstrated that the ingestion of 2 g of dileucine resulted in a rapid, distinct rise in plasma dileucine concentrations with matching leucine curves [9]; crucially, this was accompanied by an enhanced stimulation of myofibrillar protein synthesis rates relative to an equivalent amount of free leucine [9]. Indeed, the acute myofibrillar protein synthetic responses demonstrated individual variability in response to acute dileucine supplementation in this study [9]. However, this aligns with other dietary supplementation regimes where unsupervised machine learning techniques have demonstrated that individuals often partition into unique sub‐groups based on their capacity to metabolize and respond to specific nutritional strategies (i.e., personalized nutrition concepts) [10]. In any case, consistent with these acute metabolic observations, a 10‐week resistance training study reported greater improvements in lower‐body strength and muscular endurance with daily supplementation of 2 g of dileucine compared with leucine and placebo in resistance‐trained males [11]. Collectively, these acute and chronic results indicate that short‐chain leucine‐containing peptides may exert anabolic effects distinct from those of free amino acids, even if the exact mechanistic basis for these effects remains to be determined.

Importantly, while free, crystalline dipeptide ingestion established the anabolic action of the intact molecule, evaluating the effects of how these short‐chain fragments are liberated and absorbed from intact, highly processed protein isolates represented the next step in our understanding. For example, the ingestion of 12.5 g of whey or soy protein, provided in either hydrolyzed or nonhydrolyzed form, has been shown to increase postprandial plasma concentrations of BCAA‐containing dipeptides, including Leu–Leu [12]. Notably, Val–Leu and Ile–Leu were significantly higher following ingestion of protein hydrolysates compared with intact proteins, whereas Leu–Leu concentrations were similar between hydrolyzed and nonhydrolyzed conditions. With respect to protein source, whey elicited higher Leu–Leu concentrations than soy. These findings suggest that postprandial responses may differ across dipeptides, potentially reflecting dipeptide‐specific stability and enzymatic degradation in vivo. While ingestion of the highly processed protein sources can influence plasma dileucine concentrations, the effect of whole‐food protein sources on dileucine concentrations remains poorly understood.

Therefore, the purpose of this study was to determine the influence of ingesting whole protein foods on modulating the postprandial rise in plasma dileucine concentrations. Based on results from isolated protein sources [12], we hypothesized that whole food ingestion would elevate postprandial plasma dileucine concentrations compared to crystalline amino acid‐enriched mixtures or carbohydrate controls. Regarding food matrix effects, we further hypothesized that the more rapidly digested low‐fat meat would elicit higher peak dileucine concentrations (C_max_) compared to high‐fat ground meat or a plant‐based meal of beans and rice. Lastly, we hypothesized that the magnitude of the plasma dileucine response would be positively associated with myofibrillar protein synthesis.

## 2 Methods

### 2.1 Participants and Ethical Approval

This study is a secondary analysis of bio‐banked samples obtained from two independent randomized controlled trials registered at ClinicalTrials.gov (NCT05876299 and NCT06781723). Participants were healthy adults (n = 23 males = 21 and females = 2; mean ± SD: age: 25 ± 4.3 y; BMI: 25 ± 2 kg·m^−2^). Table 1 details participants’ characteristics. Data were collected in the clinical research laboratory at the University of Illinois Urbana–Champaign from July 2023 to April 2025. Female participants completed trials during the assumed follicular phase (days 3–12), verified by self‐reported calendar tracking from the first day of menstruation. This specific window was selected to standardize testing conditions, given that whole body amino acid metabolism may fluctuate across distinct phases of the menstrual cycle [1]. All participants provided written informed consent. The study procedures were approved by the University of Illinois Urbana–Champaign Institutional Review Board (IRB #23257 and #240582), conducted in accordance with the Declaration of Helsinki.

**TABLE 1:** Participants characteristics.

Variables	Mean ± SD
*n*	23 (21 M / 2 F)
Age (yr)	25 ± 4.4
Body mass (kg)	77 ± 9
BMI (kg·m^−2^)	25 ± 2.4
LBM (kg)	56 ± 8.8
Body fat (%)	26 ± 4.2
Systolic B. Pressure, mmHg	116 ± 12.6
Diastolic B. Pressure, mmHg	67 ± 9.8
Fasting plasma glucose (mg/dl)	89.8 ± 7.7
EI (kcal/d)	1792 ± 464
Protein intake (g/d)	104 ± 39
Carbohydrate intake (g/d)	165 ± 56
Fat intake (g/d)	76 ± 28
IPAQ	3934 ± 3482
GSLTPAQ	46 ± 20

Data are mean ± SD; SD, standard deviation; GSLTPAQ, Godin‐Shepherd Leisure‐Time Physical Activity Questionnaire; IPAQ, International Physical Activity Questionnaire.

### 2.2 Experimental Trials

All trials were conducted at ∼0700 h after an overnight fast of at least 10 h. Upon arrival, an antecubital venous catheter was placed for arterialized blood sampling. Serial blood samples were collected before and after treatment ingestion to assess plasma dileucine and leucine concentrations, and muscle biopsies were obtained to determine myofibrillar fractional synthesis rate (FSR) (Figure 1). Prior to treatment ingestion, participants completed a standardized bout of resistance exercise, consisting of 1 set of 10 repetitions at 65% of 10RM and 4 sets of 10 repetitions at 90% of 10RM for both the leg press and leg extension exercises, as previously described [13].

**FIGURE 1:**
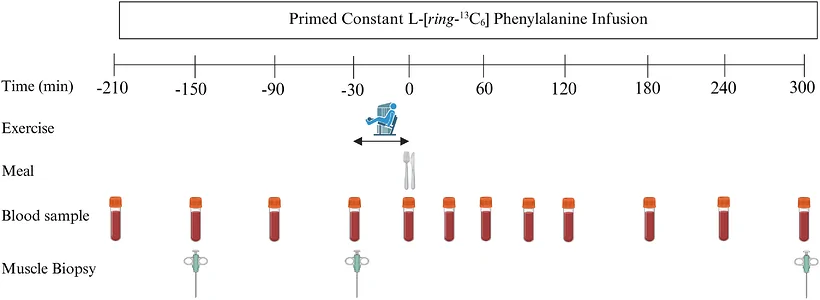
Schematic of the experimental design. Created in BioRender. Irwin, G. (2026) https://BioRender.com/cc8f85d.

### 2.3 Treatments and Food Preparation

The treatment conditions consisted of low‐fat ground pork (n = 7), high‐fat ground pork (HFP) (n = 8), plant‐based whole foods (beans and rice) (n = 8), an isolated nutrient beverage (n = 8), and a carbohydrate control (n = 8). The ground pork was oven‐cooked at 200°C until an internal temperature of 75°C was reached. Long‐grain jasmine rice (Iberia Foods, Miami, FL, USA) and canned black beans (Bush's, Knoxville, TN, USA) were used for the complementary plant‐based protein condition (COMP). The carbohydrate control (CHO) was energy‐matched to HFP using a commercially available carbohydrate beverage (Gatorade; PepsiCo, Harrison, NY, USA) prepared in 600 mL of water on the day of the trial. The isolated nutrition condition (ISO) was formulated to match the amino acid profile, carbohydrate, fat, and fiber content of the COMP condition. ISO ingredients included crystalline amino acids (Ajinomoto, Raleigh, NC, USA), maltodextrin (Nutricost, Vineyard, UT, USA), cellulose (Vital Nutrient, Middletown, CT, USA), pectin (Hoosier Hill Farm Fort Wayne, IN, USA), and soy oil (Arawana Brand, Shanghai, China). The ISO mixture was dissolved in ∼250 mL of water prior to ingestion. Participants consumed all treatments as quickly and comfortably as possible. Macronutrient and amino acid composition of uncooked pork, rice, and black beans were determined using standard AOAC methods [14]. The energy content and macronutrient composition of the five experimental treatments are shown in Table 2.

**TABLE 2:** Nutritional content of treatments.

	ISO	COMP	CHO	HFP	LFP
Amount (g)	143	251	76.6	109.6	90.2
Crude Protein (g)	20	20	/	20	20
Carbohydrates (g)	114	114	73.3	/	/
Crude Fiber (g)	2.1	2.1			
Crude Fat (g)	0.83	0.83	/	20.6	4.4
Energy (kcal/kJ)	548/2293	548/2293	266/1113	266/1113	120/502
Amino acid content					
Essential Amino Acids (g0					
Histidine (g)	0.50 (63%)	0.50 (63%)	/	0.73	0.74
Isoleucine (g)	0.94 (58%)	0.94 (58%)	/	0.99	0.92
Leucine (g)	1.62 (52%)	1.62 (52%)	/	1.54	1.46
Lysine (g)	1.05 (44%)	1.05 (44%)	/	1.75	1.68
Methionine (g)	0.35 (44%)	0.35 (44%)	/	0.52	0.51
Phenylalanine (g)	1.11 (55%)	1.11 (55%)	/	0.79	0.74
Threonine (g)	0.73 (61%)	0.73 (61%)	/	0.87	0.85
Tryptophan (g)	0.14 (42%)	0.14 (42%)	/	0.27	0.25
Valine (g)	1.14 (55%)	1.14 (55%)	/	1.03	0.96
∑ EAAs (g)	7.57 (54%)	7.57 (54%)	/	8.53	8.14
Nonessential Amino Acids					
Alanine (g)	0.93	0.93	/	1.08	0.99
Arginine (g)	1.25	1.25	/	1.24	1.12
Aspartic Acid (g)	2.00	2.00	/	1.79	1.72
Cysteine (g)	0.32	0.32	/	0.19	0.18
Glutamic Acid (g)	3.29	3.29	/	2.56	2.64
Glycine (g)	0.79	0.79	/	1.08	0.86
Hydroxylysine (g)	0.03	0.03	/	0	0
Hydroxyproline (g)	0.00	0.00	/	0.19	0.1
Lanthionine (g)	0.00	0.00	/	0	0
Ornithine (g)	0.00	0.00	/	0.05	0.04
Proline (g)	0.83	0.83	/	0.85	0.72
Serine (g)	0.96	0.96	/	0.64	0.64
Taurine (g)	0.24	0.24	/	0.06	0.04
Tyrosine (g)	0.55	0.55	/	0.88	0.88
∑NEAAs (g)	11.21	11.21	/	10.66	10.01
Leucine by total AA (%)	8.64	8.64	/	8.06	8.07
∑ BCAAs (g)	3.70	3.70	/	3.58	3.35
∑Total (g)	18.78	18.78	/	19.19	18.15

AA, amino acids; CHO, carbohydrate beverage; COMP, complementary pairing of rice and black beans. The COMP consisted of 143 g uncooked black beans and 108 g uncooked white rice, providing 10 g of protein from each source; BCAAs, branched‐chain amino acids; EAAs, essential amino acids; HFP, high‐fat ground pork; ISO, isolated mixture of crystallized amino acids, maltodextrin, cellulose, pectin, and soy oil; LFP, low‐fat ground pork; NEAA, nonessential amino acids.

### 2.4 Plasma and Muscle Analyses

Plasma samples were thawed, and 2 µL aliquots were deproteinized with 193 µL of a ddH_2_O:acetonitrile:isopropyl alcohol mixture (2:3:3, v/v/v) containing 5 µL of DL‐p‐chlorophenylalanine (0.01 mg·mL^−^^1^) as an internal standard. Samples were centrifuged at 20,817 g for 10 min at 4°C, after which 100 µL of supernatant was transferred to a new tube, dried under vacuum, and reconstituted in 100 µL of 0.1% formic acid for analysis. Plasma amino acid concentrations (limit of quantification: 2–2000 ng·mL^−^^1^) were determined by liquid chromatography–tandem mass spectrometry (LC–MS/MS) using a 5500 QTRAP mass spectrometer (SCIEX, Framingham, MA, USA) coupled to a 1290 Infinity II LC system (Agilent Technologies, Santa Clara, CA, USA). Chromatographic separation was performed on a Hypersil GOLD C18 column (2.1 × 150 mm, 1.9 µm; Thermo Fisher Scientific, Waltham, MA, USA) using mobile phase A (0.1% formic acid in water) and mobile phase B (0.1% formic acid in acetonitrile) at a flow rate of 0.45 mL·min^−^^1^. The autosampler and column temperatures were maintained at 4°C and 50°C, respectively, with an injection volume of 0.5 µL. Mass spectra were acquired in positive electrospray ionization mode, and calibration curves were generated over a concentration range of 0.05–5 µg·mL^−^^1^. Data acquisition and analysis were performed using Analyst software (version 1.7).

Myofibrillar protein‐enriched fractions were extracted from ∼50 mg of wet muscle using an ice‐cold lysis buffer (Pierce RIPA Buffer; ThermoFisher Scientific, Waltham, MA, USA; 10 µL·mg^−1^). Myofibrillar proteins were isolated by differential centrifugation and analyzed for ^13^C phenylalanine enrichments as described previously [15]

### 2.5 Reference Dileucine Data

For retrospective comparison, we used data from the dileucine arm of a prior independent trial (NCT03952884) involving ten healthy men [9]. In this referenced study, participants consumed 2 g of dileucine (DILEU), and plasma concentrations were assessed over 180 min. Due to inter‐study variations in analytical platforms that influence absolute quantification, we normalized the dataset by analyzing only the incremental area under the curve (iAUC). This approach allowed a reference assessment of total exposure under whole‐food conditions while mitigating potential baseline or calibration offsets between assays.

### 2.6 Calculations

Net incremental area under the curve (iAUC), representing the integrated change in plasma concentrations above and below baseline, was calculated for plasma dileucine and leucine concentrations over 0–60, 0–180 and 0–300 min of the postprandial period using the trapezoidal method, with baseline values defined at *t =* 0 min [16]. Peak concentration (C*max*) were calculated for all plasma concentration time curves. Previously published fractional synthesis rates (FSR; %·h^−^^1^) used for correlation analyses between dileucine and leucine Cmax were collated from the parent studies.

### 2.7 Statistical Analyses

All data are presented as mean ± standard deviation (SD) and/or mean difference (95% confidence interval [CI]). Differences in blood variables were analyzed using linear mixed‐effects models with time and condition as fixed factors and the participant‐specific random intercept. Plasma dileucine and leucine iAUC were calculated for the postprandial period, and differences were determined using linear mixed‐effects models with the condition as a fixed factor and the participant‐specific random intercept. Bonferroni's post hoc test was conducted when a significant main effect or interaction was identified. Associations between changes in myofibrillar protein synthesis rates and plasma dileucine and leucine C_max_ were examined using Pearson's correlation coefficients. For the purpose of association analysis the dileucine and leucine Cmax data were log^10−^transformed to improve normality and reduce heteroscedasticity. Statistical analyses were performed on the transformed values. The level of statistical significance was set at *p* < 0.05 for all analyses. Normality was assessed via visual inspection of normal Q‐Q plots and skewness/kurtosis values. Missing data were accommodated within the linear mixed‐effects modeling framework. Data completeness was high, with only 2% of observations missing, all of which were plasma measurements. All analyses were performed using IBM SPSS Statistics for Windows (Version 29.0.2.0; IBM Corporation, Armonk, NY, USA).

## 3 Results

### 3.1 Plasma Dileucine Concentrations

Plasma dileucine concentrations were significantly increased above baseline from 90–120 min only in the LFP condition (P<0.001; Figure 2A). At these time points, dileucine concentrations in LFP were also significantly higher than in all other conditions (all *p* < 0.05). From 180 to 300 min, plasma dileucine concentrations returned to baseline and did not differ between conditions (all *p* > 0.05). Postprandial iAUC over 300 min was significantly greater in LFP compared to CHO (*p* = 0.038), with no differences observed between LFP, ISO, COMP, or HFP conditions (all *p* > 0.05; Figure 2B). Peak dileucine concentrations (C*max*) was significantly higher in LFP compared with ISO (*p* = 0.029) and CHO (*p* = 0.016), but did not differ from HFP (*p* = 0.190) or COMP (*p* = 0.074; Table 3).

**FIGURE 2:**
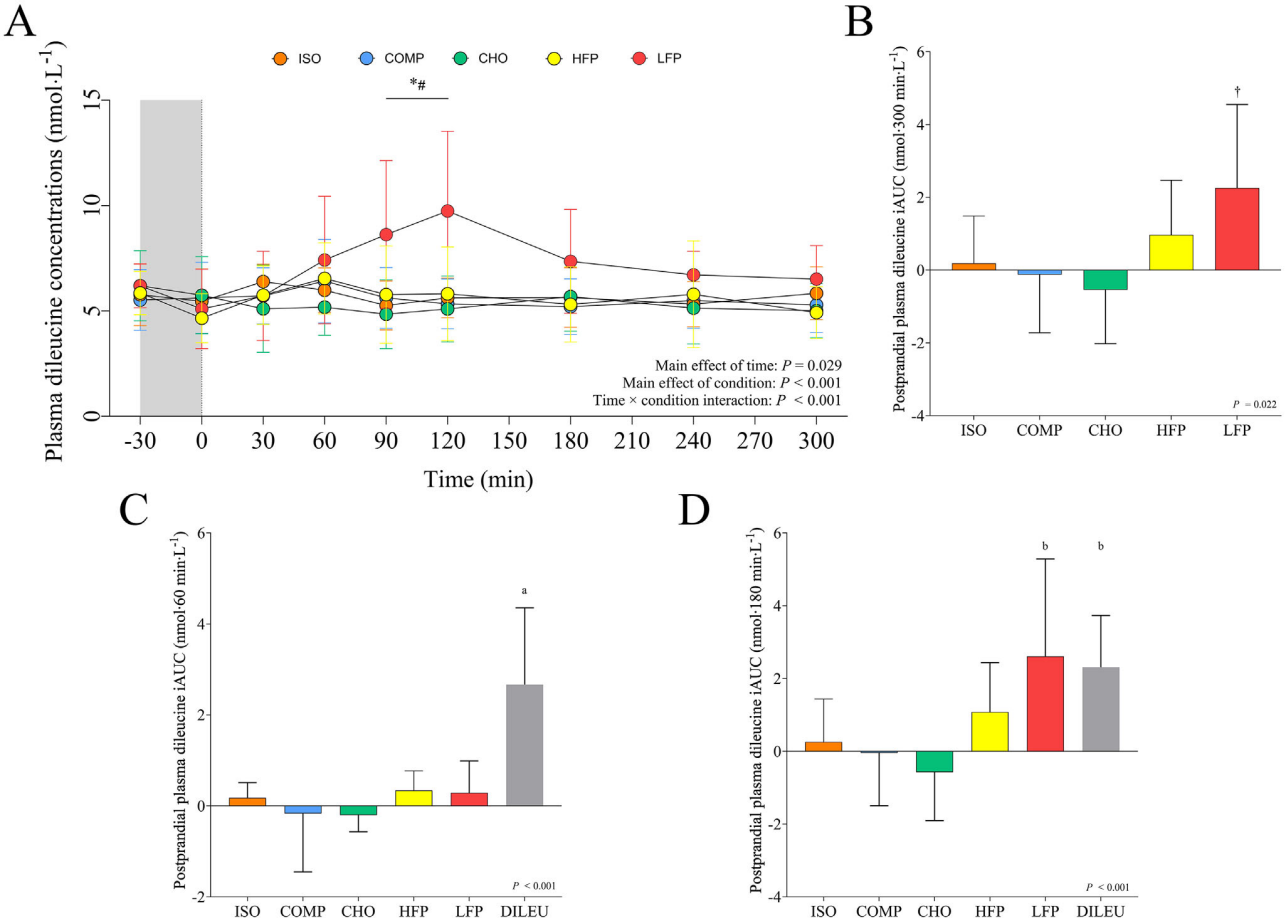
Plasma dileucine concentrations (nmol·L^−1^) before and after the ingestion of ISO (orange), COMP (blue), CHO (green), HFP (yellow), or LFP (red) (A). The gray‐ highlighted area represents the exercise bout. Data are shown as means ± SD. iAUC plasma dileucine (B) concentrations at baseline to 300 min postprandial of ISO, COMP, CHO, HFP, and LFP. iAUC plasma dileucine (C) concentrations at baseline to 60 min early postprandial of ISO, COMP, CHO, HFP, LFP, and DILEU. iAUC plasma dileucine (D) concentrations at baseline to 180 min late postprandial of ISO, COMP, CHO, HFP, LFP, and DILEU. *Denotes significantly difference from basal (t = 0; *p* < 0.05). ^#^Denotes a significant difference from LFP and other conditions (*p* < 0.05). ^†^Denotes a significant difference between LFP and CHO (*p* < 0.05). ^a^DILEU significantly (p < 0.05) different from other conditions. ^b^Denotes significant differences from CHO and COMP. CHO, carbohydrate beverage; COMP, complementary pairing of rice and black beans; DILEU, dileucine beverage; HFP, high‐fat ground pork; ISO, isolated mixture of crystallized amino acids, maltodextrin, cellulose, pectin, and soy oil; LFP, low‐fat ground pork; SD, standard deviation.

**TABLE 3:** Postprandial plasma concentration maximums (C_max_).

Variables	ISO	COMP	CHO	HFP	LFP
Dileucine (nmol·L^−1^)	6.7±0.9	7.07±1.5	6.4±1.7	7.5±2.1	10.3±3.5^b^, ^c^
Leucine (µmol·L^−1^)	185.7±23.6^a^	147±19.3	139±16.6	171.5±25.7	223.3±42.3*

Data are mean ± SD; *Significant (P < 0.05) difference between LFP, COMP, CHO, and HFP;

^a^ Significant (P < 0.05) difference between ISO and CHO;

^b^ Significant (P < 0.05) difference between LFP and ISO;

^c^ Significant (P < 0.05) difference between LFP and CHO; CHO, carbohydrate beverage; COMP, complementary pairing of rice and black beans; HFP, high‐fat ground pork; ISO, isolated mixture of crystallized amino acids, maltodextrin, cellulose, pectin, and soy oil; LFP, low‐fat ground pork; SD, standard deviation.

The retrospective comparison with the dileucine condition [9] showed a significantly greater early postprandial iAUC (0–60 min) compared with all other conditions (all *p* < 0.05; Figure 2C). Over the 0–180 min period, however, dileucine was comparable to LFP and HFP (*p* = 1.000**),** with both dileucine and LFP demonstrating greater total net exposure than COMP or CHO (*P<*0.05; Figure 2D).

### 3.2 Plasma Leucine Concentrations

A significant time × condition interaction was observed for plasma leucine (*p* < 0.001). ISO elicited a rapid peak at 30 min (*p* = 0.016; Figure 3A), while LFP and HFP resulted in delayed elevations from 60–180 min and 90–120 min, respectively. LFP maintained a significantly higher plasma leucine concentration than all other conditions during the 60–180 min postprandial window (P<0.05). In contrast, plasma leucine values fell below baseline in CHO, COMP, and ISO conditions (*p* < 0.05; Figure 3A). As such, LFP and HFP demonstrated greater 0–300 min total net exposure (iAUC) than other conditions (P<0.05). Peak leucine concentration (Cmax) was significantly higher in the LFP condition compared to COMP, CHO, and HFP (all *p* < 0.05), but did not differ from ISO (*p* = 0.153; Table 3).

**FIGURE 3:**
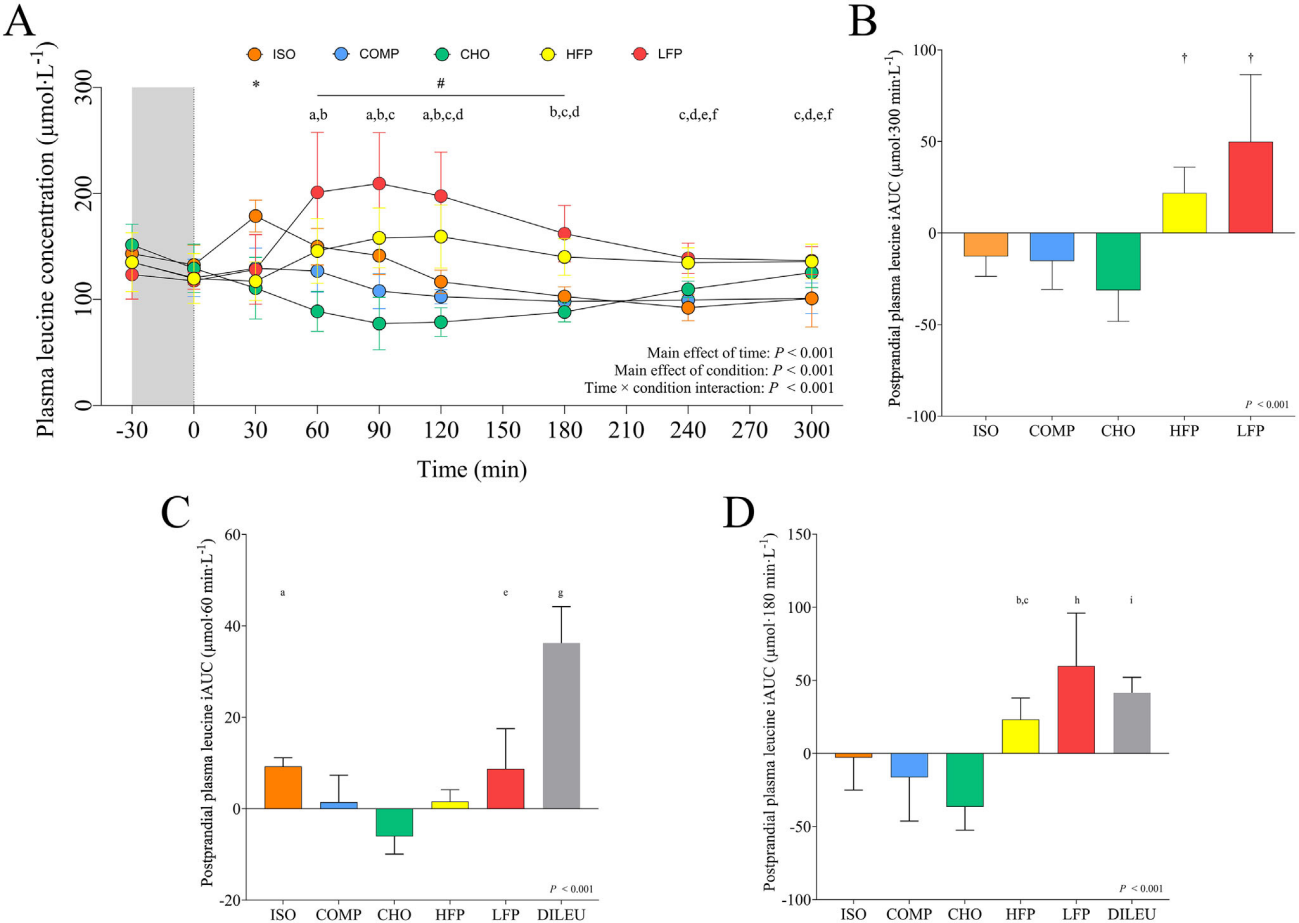
Plasma leucine concentrations (µmol·L^−1^) before and after the ingestion of ISO (orange), COMP (blue), CHO (green), HFP (yellow), or LFP (red) (A). The gray‐ highlighted area represents the exercise bout. Data are shown as means ± SD. iAUC plasma leucine (B) concentrations at baseline to 300 min postprandial of ISO, COMP, CHO, HFP, and LFP. iAUC plasma leucine (C) concentrations at baseline to 60 min early postprandial of ISO, COMP, CHO, HFP, LFP, and DILEU. iAUC plasma leucine (D) concentrations at baseline to 180 min late postprandial of ISO, COMP, CHO, HFP, LFP, and DILEU. *Denotes significant difference from ISO and other conditions (p < 0.05). #Denotes significant difference from LFP and other conditions (*p* < 0.05). ^a^Denotes significant difference from ISO to CHO. ^b^Denotes significant difference from HFP and CHO (*p* < 0.05). ^c^Denotes significant difference from HFP and COMP (*p* < 0.05). ^d^Denotes significant difference from HFP and ISO (*p* < 0.05). ^e^Denotes significant difference from LFP and ISO (*p* < 0.05). ^f^Denotes significant difference from LFP and COMP (*p* < 0.05). ^†^Significant difference from ISO, COMP and CHO (*p* < 0.05). ^g^Denotes significant difference from DILEU and other conditions (*p* < 0.05). ^h^Denotes significant difference from LFP and ISO, COMP and CHO (*p* < 0.05). ^i^Denotes significant difference from DILEU and ISO, COMP and CHO (*p* < 0.05). CHO, carbohydrate beverage; COMP, complementary pairing of rice and black beans; DILEU, dileucine beverage; HFP, high‐fat ground pork; ISO, isolated mixture of crystallized amino acids, maltodextrin, cellulose, pectin, and soy oil; LFP, low‐fat ground pork; SD, standard deviation.

In a retrospective analysis, early postprandial leucine exposure (iAUC 0–60 min) was significantly greater in the DILEU condition compared to all other conditions (all *p* < 0.001; Figure 3C). In addition, early net exposure (iAUC 0–60 min) in ISO and LFP was significantly greater than in CHO (both *p* < 0.001; Figure 3C). Over the 180 min period, LFP and DILEU resulted in the highest total leucine exposure (iAUC), significantly exceeding ISO, COMP, and CHO (all *p* < 0.05; Figure 3D). HFP was not different from LFP or DILEU and similarly exceeded CHO (*p* < 0.001) and COMP (*p* = 0.022; Figure 3D).

### 3.3 Associations Between Peak Plasma Concentrations (C*max*_)_ and Muscle Protein Synthesis

A significant moderate positive correlation was observed between peak plasma dileucine concentrations (C*max*) and the change in myofibrillar protein synthesis rates from baseline (ΔMPS) across protein conditions (r = 0.436; *p* = 0.037; Figure 4A). However, leucine C*max* of all conditions demonstrated a negligible/weak association with ΔMPS (r = 0.265; *p* = 0.102; Figure 4B). Additionally, no significant correlation was observed between cumulative dileucine exposure (iAUC) and ΔMPS across protein conditions (r = ‐0.054; *p* = 0.810; Figure 4C). In contrast, a moderate positive correlation was observed between leucine iAUC and ΔMPS across all conditions (r = 0.354; *p* = 0.032; Figure 4D). The absolute mean (±S.D.) postprandial ΔMPS for each experimental condition were as values: LFP (0.060 ± 0.029 %·h^−1^), HFP (0.028 ± 0.027 %·h^−1^), CHO (0.020 ± 0.035 %·h^−1^), COMP (0.028 ± 0.020 %·h^−1^), ISO (0.022 ± 0.023 %·h^−1^).

**FIGURE 4:**
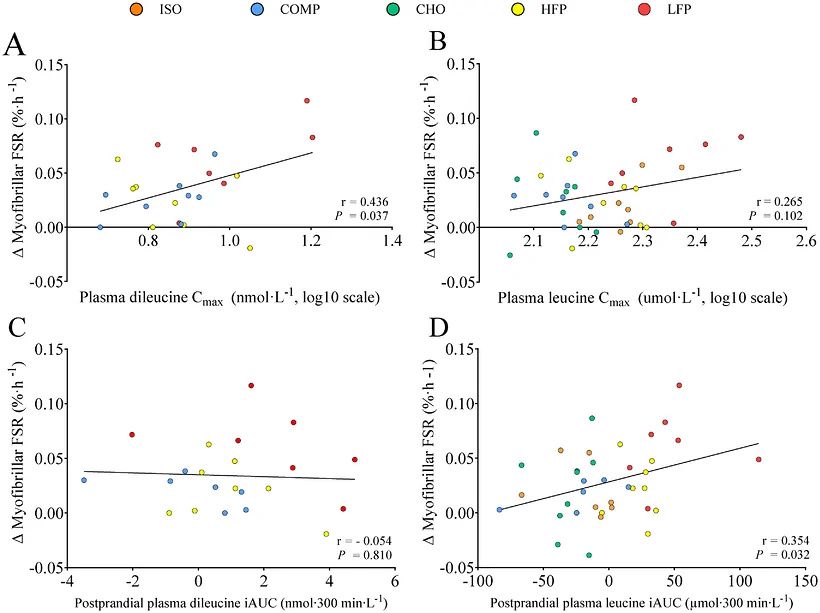
Correlation between dileucine and leucine C*max* and ∆ myofibrillar protein synthesis. Association between peak plasma dileucine C*max* for protein containing condition (µmol·L, log_10_ scale; A) and Δmyofibrillar FSR (%·h^−1^) from baseline to 300 min (r = 0.436, *p* = 0.037). Association between peak plasma leucine C*max* for all conditions (µmol·L, log_10_ scale; B) and Δmyofibrillar FSR (%·h^−1^) from baseline to 300 min. (r = 0.265, *p* = 0.102). Each point represents an individual participant. FSR, fractional synthesis rate.

## 4 Discussion

This is the first analysis to examine how ingesting animal‐ and plant‐ whole foods influences plasma dileucine concentrations and their association with post‐exercise myofibrillar protein synthesis rates in healthy young adults. We demonstrated that ingestion of moderate amounts of protein from whole‐food sources generally did not result in a robust elevation in postprandial dileucine concentrations, except for low‐fat pork (LFP). The latter also resulted in a greater postprandial leucinemia than the other treatments. Despite the relatively low plasma concentrations of dileucine compared to leucine, the peak plasma dileucine (C*max*) was positively correlated with the postprandial rise in myofibrillar protein synthesis rates (ΔMPS), whereas the leucine C*max* was not. Our results suggest that the postprandial increase in plasma dileucine after ingesting whole foods may be limited by the food matrix and digestion kinetics, at least during the post‐exercise state.

Past efforts have demonstrated that ingesting isolated proteins, such as whey or soy, increases postprandial plasma concentrations of BCAA‐containing dipeptides, such as dileucine, whether the protein is hydrolyzed or intact [12]. Notably, while protein hydrolysates yielded higher concentrations of Val–Leu and Ile–Leu compared to intact proteins, the postprandial rise in Leu–Leu concentrations were similar between the two hydrolyzed and nonhydrolyzed conditions. Regarding protein source, whey elicited higher dileucine concentrations than soy. In the current study, it is evident that the entire food matrix modulates the postprandial rise in dipeptide concentrations. Unlike isolated protein sources, proteins are entrapped within cellular structures and co‐ingested with other macronutrients in whole foods. In plant sources (e.g., COMP), proteins are encapsulated within fibrous cell walls (e.g., cellulose), whereas in intact animal food sources, proteins are bound within connective tissue. As such, depending on the specific ingested food matrix, this will differentially impact gastrointestinal transit time and the subsequent hydrolysis of dipeptides by luminal and brush‐border peptidases before they can be absorbed intact via PepT1. Furthermore, enterocytes possess substantial cytosolic peptidase activity that can rapidly hydrolyze absorbed dipeptides into their constituent amino acids prior to basolateral transport. As such, it is likely that the vast majority of dileucine within whole protein foods is cleaved into free leucine prior to entering the systemic circulation. Additionally, our previous work [9] demonstrated that elevations in plasma dileucine concentrations after supplemental dileucine ingestion do not translate into greater muscle free dileucine concentrations [6]. This suggests that dileucine may undergo hydrolysis in the bloodstream, likely due to the high specificity of leucine aminopeptidases for dileucine compared to more protease‐resistant peptides that contain proline (e.g., gly‐pro), prior to reaching skeletal muscle [17, 18, 19]. This framework also explains the absence of a detectable plasma dileucine response after CHO and ISO ingestion, which did not provide intact dietary protein substrate for dileucine cleavage. Similarly, the COMP matrix failed to elicit a significant postprandial rise in plasma leucine concentrations due to the restrictive nature of the plant matrix. This lack of hyper‐leucinemia implies that encapsulation within the structural, fibrous cell walls of the plant matrix likely generated a limited supply of leucine‐containing peptides available for PepT1‐mediated absorption. Collectively, these findings support the notion that elevations in blood dileucine after whole‐food ingestion is limited by both gastrointestinal and postprandial hydrolysis.

In this context, the divergent dileucine responses between low‐fat, HFP, and the plant‐based condition offer critical insights into the role of the whole‐food matrix. As noted, the majority of food‐derived dileucine is likely cleaved into free leucine by intestinal and circulating peptidases prior to reaching systemic circulation. It is interesting; however, that LFP specifically overcame this barrier. We speculate that meat grinding will physically disrupt the connective tissue network, favoring more rapid dietary protein digestion and amino acid absorption kinetics [20]. In contrast, co‐ingesting dietary fat and energy is known to slow gastric emptying and delay protein digestion and absorption, thereby altering the temporal pattern of dietary protein‐derived amino acid and peptide appearance rates into circulation [21]. Although total protein and leucine content were matched, the higher lipid content of HFP likely delayed the liberation and intestinal transport of short‐chain peptides such as dileucine, resulting in a lower plasma dileucine temporal response without significantly altering total net absorption compared to LFP ingestion. Because cumulative iAUC and C*max* were statistically identical between pork conditions, these profiles suggest a distinct temporal shift in the pattern of peptide appearance rather than total peptide generation, such that the more rapid delivery of peptides after LFP ingestion briefly exceeded hydrolytic capacity that manifested as a robust, measurable increase in plasma dileucine (∼90–120 min; Figure 2). Similarly, plant proteins in the COMP condition are encapsulated within fibrous cell walls, slowing digestion resulting in the dileucine being cleaved into free leucine before it could accumulate systemically. This time‐course of elevation after LFP ingestion contrasts with our previous investigation using an isolated dileucine supplement, which produced a rapid yet short‐lived peak in plasma dileucine between 15–60 min [9]. However, the similarity in overall dileucine exposure between the more readily digested LFP and free dileucine condition supports the notion that a portion of food‐derived dileucine can surpass gastrointestinal hydrolysis and appear in circulation, albeit with a delayed temporal profile after whole‐food intake.

We previously demonstrated that ingesting 2 g of dileucine elevated plasma dileucine concentrations more than an equivalent dose of free leucine, despite similar increases in plasma leucine in healthy young adults. This result was also accompanied by higher myofibrillar protein synthesis rates in the dileucine versus the leucine condition [9]. A novel observation in the present study is that we observed a positive association between peak plasma dileucine (C*max)* and the change in myofibrillar protein synthesis rates (ΔMPS) across protein conditions (Figure 4A), but not with leucine C*max*. It is important to note, however, that the association is modest (r = 0.436), and given the relatively low plasma concentrations of dipeptides, we believe this should be interpreted as hypothesis‐generating rather than causal. In particular, the moderate strength of this correlation indicates that Cmax alone explains only a modest proportion of the variance in ΔMPS and is consistent with the notion that the regulation of MPS after whole foods is nuanced and cannot be predicted by a single anabolic factor. To further elucidate if cumulative postprandial dipeptide exposure (iAUC) also shared a meaningful relationship with muscle anabolism, we correlated ΔMPS against plasma dileucine iAUC. This analysis demonstrated a completely negligible, nonsignificant association (r = ‐0.054; *p* = 0.810; Figure 4C). Hence, it appears that the anabolic action of circulating dileucine is more a function of its peak postprandial magnitude rather than the total net exposure.

Additionally, it is important to recognize that plasma dileucine C*max* functions purely as a peak static concentration snapshot, offering no insight into underlying pool kinetics. While an elevated C*max* is normally attributed to improved protein digestion kinetics and rapid intestinal absorption [22] this peak could also be, in theory, influenced by downstream changes in whole‐body clearance, such as reduced tissue uptake or blunted extracellular peptidase activity. Therefore, the observed positive association between dileucine C*max* and ΔMPS should not be misconstrued as definitive evidence that circulating dileucine directly stimulates muscle anabolism. Without ‘true’ kinetic resolution, a higher dileucine C*max* may simply serve as a passive surrogate marker of favorable digestive kinetics and subsequent nutrient availability. Moving forward, clarifying these kinetics would require the development of a novel, isotopically labeled dileucine tracer. However, total whole‐body dileucine flux remains completely unknown. So, the utility of this tracer would be limited to simply tracing the metabolic fate of the intact molecule rather than modeling more elegant multi‐pool kinetics.

In conclusion, ingestion of moderate doses of whole‐food protein sources does not appear to substantially elevate postprandial plasma dileucine concentrations, except for LFP, which elicited a detectable increase. Furthermore, a retrospective comparison with free dileucine ingestion suggested distinct kinetic differences, with whole‐food animal protein demonstrating a delayed postprandial rise in the plasma dileucine response. Yet, these findings should be interpreted with the caveat that total energy content of the meals was not matched across whole‐food conditions, with COMP matrix providing 548 kcal compared to only 120 kcal for the LFP condition. Whether the rapid, distinct plasma dileucine response observed after LFP ingestion would be maintained if it were consumed as part of a larger, energy‐matched mixed meal remains to be determined, as the additional macronutrient load would alter gastric emptying rates and intestinal peptide absorption kinetics. Additionally, we did not determine the exact timeline of dynamic, intraluminal dileucine generation from the experimental meals during digestion. This leaves open the question of whether the divergent plasma dileucine responses we observed were related to upfront differences in peptide liberation within the gut or how rapidly they are broken down inside the intestinal cells. Nevertheless, because we matched the total protein dose at 20 g across the whole food conditions, the substrate pool available for digestion was identical. This indicates that the differential plasma dileucine responses were more likely attributable to food matrix‐dependent differences in digestion and the capacity of liberated peptides to rapidly flood and saturate the low‐affinity, high‐capacity PepT1 system, thereby allowing intact dipeptides to escape splanchnic clearance and reach the circulation, rather than differences in total protein intake. Future studies are warranted to quantify intraluminal peptide generation and track these short‐chain fragments in vivo. This will help unravel the mechanisms governing food‐derived dileucine formation, intestinal survival/absorption, and its ultimate contribution to post‐exercise MPS responses.

## Funding

This work was funded by the Pork Checkoff (PR‐005301) and Hatch Act of 1887 (project award number 7007302), from the U.S. Department of Agriculture's National Institute of Food and Agriculture. The sponsor was only involved in financial support of the project, without involvement in design, data collection, and analysis, nor interpretation and dissemination of the report.

## Clinical Trial Registry

ClinicalTrials.gov (NCT05876299 and NCT06781723).

## Conflicts of Interest

The authors declare no conflicts of interest.

## Data Availability

Data described in the manuscript, code book, and analytic code will be made available upon request pending approval from the principal investigator.
